# Assessment of bone densitometry in Iranian patients with multiple sclerosis: A case-control study

**Published:** 2013

**Authors:** Mehdi Moghaddasi, Mahbubeh Aghaei

**Affiliations:** Department of Neurology, Rasool-e-Akram Hospital AND Iranian Center of Neurological Research, Tehran University of Medical Sciences, Tehran, Iran

**Keywords:** Relapsing-Remitting Multiple Sclerosis, Interferon, Bone Mineral Densitometry

## Abstract

**Background:**

We compared bone mineral density (BMD) in patients with relapsing-remitting multiple sclerosis (RRMS) on interferon with that of patients with relapsing-remitting multiple sclerosis (RRMS) who were not receiving interferon and healthy age- and sex-matched controls.

**Methods:**

Overall, 30 patients with RRMS on interferon (treated patients), 30 patients with RRMS but not receiving interferon (untreated patients), and 30 healthy controls were enrolled. The subjects were matched for age, sex, body mass index, physical activity and nutritional habits (as possible), duration of illness, frequency of attacks, and the amount of corticosteroid therapy. BMD was measured at the lumbar spine and proximal femur. The results of dual-energy X-ray absorptiometry were expressed as BMD (g/cm^2^), Z-scores, and T-scores.

**Results:**

Osteopenia in patients with RRMS was 61.7% in proximal femur and 53.3% in lumbar spine (vs. 53.3% and 40% in healthy controls, respectively). There was an inverse relationship between Expanded Disability Status Scale scores and lumbar and femoral BDM in the patients. In treated patients, there was an inverse relationship between the duration of interferon therapy and lumbar and femoral BDM. In untreated patients, there was a similar relation between the duration of the illness and BMD. Moreover, inverse relationships existed between the frequency of attacks and lumbar and femoral BDM in both treated and untreated groups. However, this association was only significant in the untreated group.

**Conclusion:**

Patients with MS showed reduced BMD in comparison with healthy controls. This reduction was related to the frequency of attacks. We also found lower BMD in untreated patients compared to interferon-treated patients.

## Introduction

Multiple sclerosis (MS) is an inflammatory autoimmune disease of the central nervous system (CNS) which manifested as acute focal inflammatory demyelinization and axonal loss; it is one of the most important causes of neurological disability in young adults.^1^ The course of the disease may be progressive or relapsing-remitting, and its disabilities may have a detrimental effect on the ambulatory status and physical activity of the patients. Its severe disabilities can lead to progressive immobilization.^1–3^

Treatment with corticosteroids for acute attacks or as an intermittent continuous therapy, in combination with immobility, malnutrition, low exposure to sunlight, leading to deficiency of vitamin D and genetic factors can lead to osteoporosis.^2, 4–6^

A limited number of studies have shown that MS patients have significantly reduced bone mineral density.^2, 4–6^ Little is known about the effect of immunomodulatory therapy (IMT) on bone mineral density (BMD) in MS. Reviews on MS do not mention anything about the effect on bone of IMT.^7, 8^

The aim of this study was to assess bone mineral density in patients with relapsing-remitting multiple sclerosis (RRMS) who were treated with interferons in comparison to patients with RRMS who did not get interferon and healthy age- and sex-matched controls.

## Materials and Methods

### Study population

This case control study was conducted at Rasool Akram hospital affiliated to Tehran University of Medical Sciences, Tehran, Iran. The institutional medical ethics committee approved the study and all patients and controls enrolled after signing their written informed consents. Thirty patients with RRMS who were treated with interferon (treated patients), 30 patients with RRMS who were not treated with interferon (untreated patients), and 30 healthy controls who were referred to neurology clinic, were enrolled. The diagnosis of RRMS was based on revised McDonald criteria diagnostic scheme.^9^ All of the participants in this investigation were matched by age, sex, body mass index (BMI), physical activity (as possible), nutritional habits (as possible), duration of illness, frequency of attacks and the amount of corticosteroid therapy. Patients with endocrinopathies, rheumatologic disorders, liver and renal failure, users of supplementations and patients who were treated with oral corticosteroid therapy were excluded. For more equalization, serum calcium, phosphorus, parathyroid hormone (PTH), 25 (OH) vitamin D3 and alkaline phosphatase levels were also matched. All participants were interviewed by one neurologist. The demographic, past medical history, family history and medication history were questioned. Patient's disability score was estimated according to Extended Disability Status Scale (EDSS).

### Bone mineral densitometry

Bone mineral density was measured at the lumbar spine and proximal femur, using dual-X-ray absorptiometry (DXA), on a Lunar DPX densitometer (Lunar, Madison, WI, USA). Values for results of DXA measurements were expressed as BMD (g/cm^2^), Z scores and T-scores.

### Statistics

The data was expressed as mean ± standard deviation (SD). Chi-square and Student's t tests were used for qualitative and quantitative data, respectively. We used Pearson's correlation analysis for assessing correlation between EDSS, 25 (OH) vitamin D3 levels, BMI, duration of treatment with interferon, T-score, and Z-score. We considered P < 0.05 as statistically significant. All statistical analyses were performed with SPSS software version 16 (SPSS Inc. Chicago, IL, USA).

## Results

A total of 30 treated RRMS patients, 30 untreated RRMS patients and 30 healthy controls entered the study. Baseline characteristics of the subjects are listed in Table 1. There were no significant differences in serum calcium, phosphorus, PTH, 25 (OH) vitamin D3 and alkaline phosphatase levels between 3 groups (Table 2).

**Table 1:** Demographic features of subjects in three study groups

	Age (year) (min-max)	BMI (kg/m^2^)	Duration of illness (month)	Attack frequency	Methylprednisolone pulse therapy (g)	EDSS
IFN treated RRMS	20.8 ± 6.6 (13-42)	24.6 ± 3.9 (19.3-33.2)	56.5 ± 39.3 (7-132)	3.44 ± 1.99 (1-12)	12.2 ± 11.2 (0-60)	2.76 ± 0.92 (1.5-5.5)
Untreated RRMS	30.6 ± 7.7 (19-48)	24.16 ± 4.0 (17.2-32)	36.3 ± 43.5 (6-180)	2.9 ± 1.99 (1-12)	8.96 ± 11.4 (0-60)	2.5 ± 0.96 (1-4.5)
Healthy controls	33.0 ± 8.2 (22-45)	23.8 ± 4.9 (32.8-16.4)				
P	0.11	0.79	0.09	0.60	0.28	0.27

BMI: Body mass index, EDSS: Expanded Disability Status Scale, INF: Interferon; RRMS: Relapsing remitting multiple sclerosis

**Table 2:** Serum calcium, phosphorus, PTH, 25 (OH) vitamin D3 and alkaline phosphatase levels in three study groups

	Calcium (mg/dl) (min-max) (8.5-11)	Phosphorus(mg/dl) (min-max) (2.5-5)	25(OH)vitD3 (ng/ml) (min-max)*	PTH (pg/ml) (min-max) (15-65)	Alk-Ph (Iu/ml) (min-max) (64-306)
IFN treated RRMS	8.8 ± 0.8 (8.1-10.4)	4.01 ± 0.77 (2.9-5.2)	25.6 ± 16.3 (6-58)	31.1 ± 16.4 (10-66)	110.4 ± 35.3 (64-274)
Untreated RRMS	8.84 ± 0.73 (8.1-10.9)	3.76 ± 0.56 (3.1-5)	44.3 ± 62.2 (4.1-234)	30.4 ± 7.8 (18-56)	93.7 ± 22.7 (67-140)
Healthy control	9.2 ± 0.66 (8.4-10.9)	3.72 ± 0.59 (3-5.2)	35.2 ± 19.7 (15-86)	34.3 ± 13.7 (18-56)	89.3 ± 12.3 (75-118)
P	0.22	0.48	0.43	0.68	0.07

* Deficient: < 10, Insufficient: 10-29, Sufficient: 30-100, Potential intoxication: > 100

PTH: Parathyroid hormone, Alk-Ph: Alkaline phosphatase, IFN: Interferon, RRMS: Relapsing remitting multiple sclerosis

**Figure 1:**
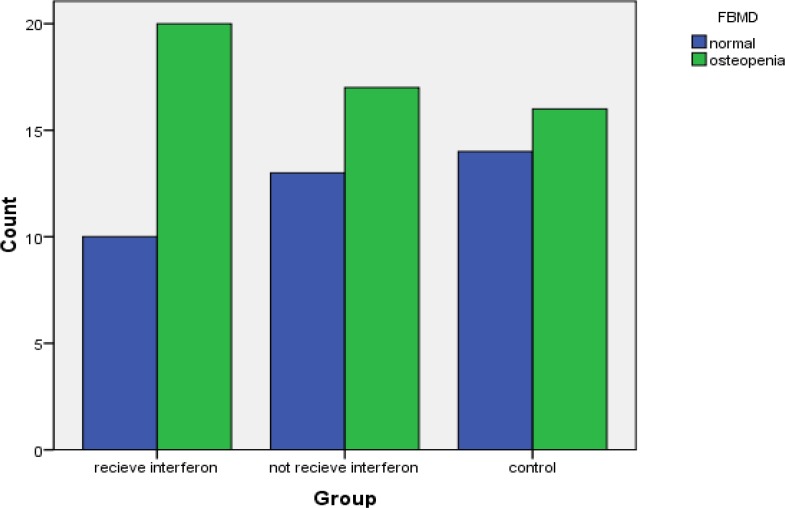
The frequency of normal and osteopenic bone mineral densitometry in femoral region in interferon (INF) treated bone mineral density (RRMS) and untreated RRMS patients and controls

**Figure 2:**
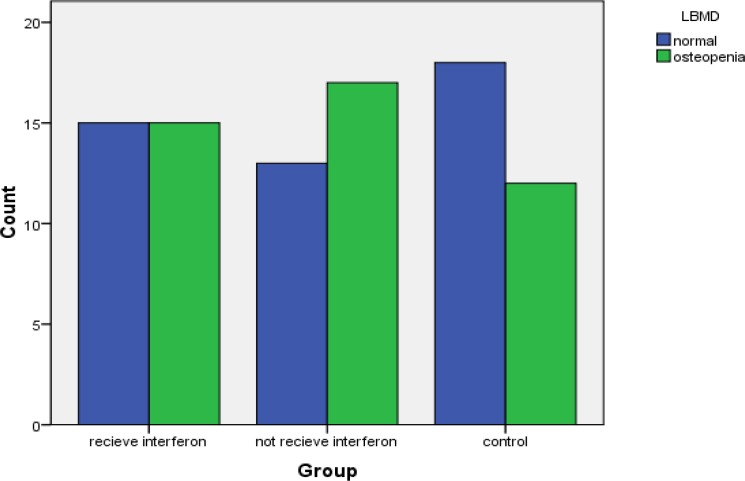
The frequency of normal and osteopenic bone mineral densitometry in lumbar region in interferon (INF) treated bone mineral density (RRMS) and untreated RRMS patients and controls

Frequency of osteopenia in femoral BMD in RRMS patients (regardless of treatment) was 61.7% (vs. 53.3% in healthy controls) and in lumbar BMD was 53.3% (vs. 40% in healthy controls) (Figures 1 and 2). The T-scores, Z-scores and BMD status of femoral and lumbar regions are presented in Table 3.

**Table 3:** Difference between lumbar and femoral bone mineral densitometry in three groups

	Lumbar BMD (min-max)			Femoral BMD (min-max)			Densitometry conclusion					

							Lumbar BMD			Femoral BMD		

	Z-score	T-score	g/cm^2^	Z-score	T-score	g/cm^2^	Normal	Osteopenia	Osteoporosis	Normal	Osteopenia	Osteoporosis
IFN treated RRMS	-0.81 ± 1.04 (-2.4 – 1.6)	-0.89 ± 1.08 (-2.3 – 1.6)	3.04 ± 11.30 (0.68-61.82)	-0.89 ± 0.96 (-2.3 – 1.2)	-1.03 ± 0.9 (-2.3 – 1.2)	0.87 ± 0.67 (0.6-4.31)	15 (50%)	15 (50%)	0	10 (33.3%)	20 (66.7%)	0
Untreated RRMS	-1.05 ± 0.93 (-2.6 -0.8)	-1.1 ± 0.88 (-2.7 – 0.5)	0.93 ± 0.09 (0.75-1.1)	-0.83 ± 0.93 (-2.3 – 1.3)	-1 ± 0.93 (-2.4 – 1)	0.76 ± 0.12 (0.1-1)	13 (43.3%)	17 (56.7%)	0	13 (43.3%)	17 (56.7%)	0
Healthy control	-0.62 ± 0.89 (-2.1 -1.4)	-0.79 ± 0.84 (-2 – 1.4)	1.03 ± 0.26 (0.85-1.96)	-0.78 ± 0.73 (-2 – 0.7)	-1.02 ± 0.77 (-2.2 – 0.7)	0.85 ± 0.26 (0.6- 1.72)	18 (60%)	12 (40%)	0	14 (46.7%)	16 (53.3%)	0
P	0.23	0.24	0.50	0.89	0.98	0.30	0.43	0.55				

BMD: Bone mineral densitometry, IFN: Interferon, RRMS: Relapsing remitting multiple sclerosis

In RRMS patients there was a significant increase in lumbar and femoral Z-scores and T-scores with increment in BMI (r = 0.5, P = 0.001 for untreated patients), (r = 0.3, P = 0.04 for INF treated patients).

There was reverse association between EDSS and lumbar and femoral Z-scores and T-scores in untreated (r = -0.01, P = 0.2) and INF treated RRMS patients (r = - 0.2, P = 0.6).

In INF treated RRMS patients, there was a reverse association between duration of INF therapy and lumbar (r = -0.008, P = 0.9) and femoral (r = -0.1, P = 0.5) Z-scores and T-scores.

In untreated RRMS (r = 0.6, P = 0.1), INF treated RRMS patients (r = 0.11, P = 0.7), and healthy controls (r = 0.2, P = 0.2) there was no significant increment in lumbar and femoral Z-scores and T-scores with increment in serum levels of 25 (OH) vitamin D3.

In untreated RRMS patients there was a reverse association between duration of illness and BMD (r = -0.3, P = 0.07). Although there was a reverse association between frequency of attacks and lumbar and femoral Z-scores and T-scores in both treated (r = -0.3, P = 0.08) and untreated groups (r = -0.4, P = 0.02), this association was significant in untreated group.

## Discussion

In healthy individuals, bone mass increases until 25 to 30 years of age, followed by an age-related decline in bone mineral density (BMD) from approximately 40 years of age in both sexes.^10^ Approximately, 70% of the variations in peak bone mass are determined by heredity. The remaining 30% to 40% of variability is related to environmental factors. The main factors which effect BMD are levels of calcium and vitamin D, physical activity, hormonal status and chronic diseases and medications.^11^ Data from several studies suggest that changes in BMD occur at a younger age in women and men with MS than in the general population.^12^ In patients with MS, limited physical activity due to the disabilities may interfere with bone mass acquisition and cause osteopenia and osteoporosis earlier than in healthy individuals. Studies show that BMD at the femoral neck decreases with increasing MS-related disability (EDSS-score).^12^

In our study, there was evidence of reduced BMD in MS patients (INF treated and untreated) in comparison with healthy control. A large retrospective study of 9029 MS patients in the USA, found that 15.4% MS patients have osteoporosis.^13^

The inflammatory process of MS may be responsible for the reduction in BMD. Osteoclast differentiation is derived by a factor called receptor activator of nuclear factor kappa B ligand (RANKL)^14^ and significantly higher levels of serum RANKL and osteoprotegrin (OPG) levels were found in the MS patients.^15^

The results of this study suggested that there was a reverse association between EDSS, lumbar and femoral BMD. Reduced physical activity results in induction of osteoclast activity and bone loss with a reduction of BMD, a process called immobilization or disuse osteoporosis.^16–18^

A significant correlation between disease duration and hip BMD has been established.^19^ In our study, we suggested that the higher attack frequency in MS patients can be associated with lower BMD. Any factors that effect on disease progression, disability and attack frequencies can effect on BMD. We showed that there was lower BMD in untreated MS patients in comparison with INF treated patients. Although this difference was not significant, but in longer duration INF therapy can influence BMD. Theoretically, interferon beta (IFNB) should have a beneficial effect on bone. Osteoclasts differentiation is mediated by RANKL (receptor activator of nuclear factor kappa beta ligand) signaling.

Takayanagi et al.^20^ showed that RANKL induced the IFNB gene in osteoclast precursor cells, and subsequently IFNB inhibited the differentiation of osteoclasts for auto regulation. Weinstock-Guttman et al.^21^ reported that in-vitro treatment of peripheral blood lymphocytes with IFNB reduced osteoclastogenesis in response to RANKL. These insights add meaning to the apparent favorable effect of IFNB on bone that we noted in our study. Shuhaibar et al. also, have reported that both mean BMD Z-score at spine and mean BMD Z-score at femur were significantly greater than zero.^22^ Kampman et al. have shown that measured Z-scores did not differ between IMT-treated and untreated patients.^23^

These differences between studies may be due to differences in the number of patients, duration of illness, differences of other factors which affected BMD such as nutrition, sun exposure and physical activities.

Kampman et al. have suggested a protocol for measurement of BMD in patients with MS. They suggested that measure bone mineral density (BMD) within a couple of years after diagnosis of MS and repeat BMD measurements after 3–5 years in patients with normal bone density.^11^

## Conclusion

We suggested that since patients with MS are at higher risk of falling due to disabilities early diagnosis of bone loss, early treatment can reduce the morbidity and mortality of osteoporosis in these patients.
